# Immunogenetic effects of low dose (CEM43 30) magnetic nanoparticle hyperthermia and radiation in melanoma cells

**DOI:** 10.1080/02656736.2019.1627433

**Published:** 2019-11

**Authors:** Kayla E. A. Duval, Nicholas A. Vernice, Robert J. Wagner, Steven N. Fiering, James D. Petryk, Gabriela J. Lowry, Steven S. Tau, John Yin, Georgia R. Houde, Aneeq S. Chaudhry, P. Jack Hoopes

**Affiliations:** a Thayer School of Engineering, Dartmouth College, Hanover, NH, USA; b Geisel School of Medicine, Dartmouth College, Hanover, NH, USA

**Keywords:** Magnetic nanoparticle hyperthermia, radiation, apoptosis, cytokines, immunology

## Abstract

**Objective:**

In this in vitro study we have used an RNA quantification technique, nanoString, and a conventional protein analysis technique (Western Blot) to assess the genetic and protein expression of B16 murine melanoma cells following a modest magnetic nanoparticle hyperthermia (mNPH) dose equivalent to 30 minutes @ 43°C (CEM43 30) and/or a clinically relevant 8 Gy radiation dose.

**Methods:**

Melanoma cells with mNPs(2.5 μg Fe/106 cells) were pelleted and exposed to an alternating magnetic field (AMF) to generate the targeted thermal dose. Thermal dose was accurately monitored by a fiber optic probe and automatically maintained at CEM43 30. All cells were harvested 24 hours after treatment.

**Results:**

The mNPH dose demonstrated notable elevations in the thermotolerance/immunogenic HSP70 gene and a number of chemoattractant and toll-like receptor gene pathways. The 8 Gy dose also upregulated a number of important immune and cytotoxic genetic and protein pathways. However, the mNPH/radiation combination was the most effective stimulator of a wide variety of immune and cytotoxic genes including HSP70, cancer regulating chemokines CXCL10, CXCL11, the T-cell trafficking chemokine CXCR3, innate immune activators TLR3, TLR4, the MDM2 and mTOR negative regulator of p53, the pro-apoptotic protein PUMA, and the cell death receptor Fas. Importantly a number of the genetic changes were accurately validated by protein expression changes, i.e., HSP70, p-mTOR, p-MDM2.

**Conclusion:**

These results not only show that low dose mNPH and radiation independently increase the expression of important immune and cytotoxic genes but that the effect is greatly enhanced when they are used in combination.

## Introduction

For many types of cancer, individual therapies rarely result in a cure. Therefore, the use of adjuvant-based combinatorial treatments centered around surgery, radiation and chemotherapy have become standard practice. Many of these adjuvant therapies, including hyperthermia, immunotherapy and photodynamic therapy, have been shown to work well in combination with the three primary treatment modalities [1-3]. However, in spite of thousands of positive preclinical research studies demonstrating the cancer treatment efficacy of these combination treatments, clinical trials have often failed to reach the predicted or anticipated positive clinical result. As the underlying mechanism by which clinical level hyperthermia affects living cells, alone or in combination with other modalities, remains challenging in many settings, achieving a consistent positive therapeutic effect has proven difficult. Researchers and clinicians who work in this field, believe there are two major reasons for this unfulfilled clinical promise; first that the intersection of tumor, normal tissue and hyperthermia biology is variable, inconsistent and complex in various individual treatment situations and second that current thermal dose delivery technology is not precise or selective enough, especially regarding individual cancer cell cytotoxicity or sensitization. Without such precisely targeted heating technology and detailed mechanistic tumor/normal tissue biology information, at least for invasive cancers, one strategy for advancing hyperthermia cancer treatment success is to use cell-based nanoparticle heating techniques and sublethal tumor heating to stimulate molecular anticancer immune and/or cytotoxic mechanisms such as apoptosis. This *in vitro* study, using melanoma cells, magnetic nanoparticle hyperthermia (mNPH), quantitative RNA genetic analysis (nanoString) and semi-quantitative protein analysis (Western blot) was designed to better understand, at the genetic and protein level, if low-dose hyperthermia alone or with modest radiation is capable of stimulating a meaningful tumor cell based immune and/or cytotoxic response. These *in vitro* studies do not recapitulate an *in vivo* setting. So, the results here do not clarify how the changes observed would influence immune reactivity *in vivo*. We believe our use of quantitative genetic analysis, in this setting, has resulted in the generation of novel cancer cell-based immunogenic and protein information, for low dose nanoparticle hyperthermia and radiation, that will allow for more specific and effective improvements in the use of these therapeutics.

## Methods

### Cell lines and growth conditions

B16-F10 murine melanoma cells were obtained from the American Type Culture Collection (ATCC) (Manassas, VA). The cells were plated at a density of 1.25 × 10^5^ cells/mL and cultured in 1× Roswell Park Memorial Institute (RPMI) with 4.5 g/L glucose and l-glutamine, without sodium pyruvate (Mediatech, Inc., Manassas, VA) supplemented with 10% fetal bovine serum (FBS) (HyClone Laboratories, Inc., Logan, UT), and 1% penicillin–streptomycin solution (10,000 units/mL penicillin; 10,000 μg/mL streptomycin, HyClone Laboratories, Inc., Logan, UT). Cells were cultured on tissue culture dishes and incubated at 37 °C and 5% CO_2_.

### Magnetic nanoparticles

All experiments were conducted with Bionized NanoFerrite (BNF) iron oxide nanoparticles (micromod Partikeltechnologie GmbH, Rostock, Germany). The dextran matrix/shell 100 ± 30 nm (hydrodynamic diameter) mNPs were suspended in H_2_O. The NPs are 75–80% (w/w) magnetite and have a stock solution concentration of 45 mg/mL (24.5 mg/mL iron). The mNPs were added to the cell media for a final concentration of 3 mg/mL and incubated for 48 h pretreatment.

### AMF activation

Following a 48 h mNPH incubation, cells were trypsinized and spun to form pellets in 1.5 mL Eppendorf tubes. Pellets contained 3 × 10^6^ cells with 2.5 pg iron/cell (~7.5 μg iron per pellet). To generate the targeted thermal dose, the pelleted cells were exposed to an alternating magnetic field (AMF) that was generated by a custom-built, water cooled 14 turn solenoid coil powered by a Huttinger TIG 10/300 generator (Freiburg, Germany). The AMF was tuned to operate at 165 kHz and 300 Oe. Cell medium and near environmental temperatures were continuously monitored and kept stable using FISO fiber optic temperature monitoring probes (FISO Inc., Quebec, Canada, operated at a frequency of 1 Hz). A thermally controlled water bath surrounded the cell tubes to maintain the environmental tube temperature at 37 °C ± 1 °C. The water bath was monitored with a separate FISO temperature probe. Following AMF exposure yielding a CEM43 30, the cells were re-plated and placed into the incubator before lysing at 24 h. All mNPH treated cells received a thermal dose of CEM43 30, cumulative equivalent minutes at 43 °C for 30 min, with stable maximum temperatures between 42 and 44 °C.

### Treatment conditions

To ensure the genetic and protein changes observed were associated with the mNPH, 8 Gy and mNPH + 8 Gy treatments, eight different treatment condition groups were studied (under identical experimental conditions). These control groups included: B16 cells+no treatment, cells+mNP, cells+AMF, cells+mNPH (mNPs+AMF), cells + 8 Gy, cells + 8 Gy+mNPs, cells + 8 Gy+AMF, cells + 8 Gy+mNPH (mNPs+AMF). All treatment groups, including controls, underwent pelleting and were placed in the water bath for the same amount of time as the groups that were activated by the magnetic field. This ensured that changes in expression were truly due to the treatments and not due to experimental procedure stress. As the genetic expression changes for all control groups (groups not exposed to mNPH and/or radiation) were not statistically different than no treatment we have not provided genetic response details. As such, the result section contains only genetic and protein data for mNPH, 8 Gy and mNPH + 8 Gy. These effects/data are compared to the no treatment group, also referred to as ‘control’.

### Radiation

Cells were irradiated using a cesium-137 irradiator at 1000 rad/minute (662 keV). Cell culture plates were given an 8 Gy dose immediately before being pelleted and placed in a temperature controlled AMF coil operated at 160 kHz and 300 Oe. 8 Gy is a regularly used clinical and research dose.

### Protein isolation and quantification

Protein for Western blot analysis was harvested with lysate buffer (40 mM Hepes buffer, 120 mM sodium chloride, 1 mM EDTA, 2.5 mM sodium pyrophosphate, 1 mM β-glycerol-phosphate, 1% Triton-X 100, 0.01% sodium deoxycholate) 24 h post treatment, isolated and normalized with the Bradford protein assay. Traditional Western blot protocol was carried out using SDS-PAGE. Briefly, 12% polyacrylamide separating gels were used with 16 μg of lysate sample loaded per well. These gels were then transferred to nitrocellulose membranes which were then incubated in protein specific antibodies. The secondary antibodies were conjugated with horseradish peroxidase allowing for immunoreactive bands to be detected using a G:Box Syngene through enhanced chemiluminescence (New England Biogroup, Atkinson, NH). Protein expression was normalized with respect to expression of GAPDH and quantified via densitometry performed with NIH ImageJ Software. Each experiment was run three times with each replicate having its own RNA and protein analysis.

### RNA collection and analysis

RNA was harvested and purified using the Qiagen RNeasy Mini Kit, according to the manufacturer’s instructions (Qiagen, Venlo, Netherlands). RNA concentration and purity were determined with the GeneQuant II spectrophotometer (Pharmacia Biotech, Piscataway, NJ). RNA samples were normalized so that 50 ng of RNA was used per sample for use with nanoString technologies for expression levels. The nanoString PanCancer Immune Profiling Panel (murine) combined with a custom nanoString nCounter panel (apoptosis, cell cycle, DNA damage genes), was used on our samples and quantified on the nanoString nCounter Analysis System (nanoString Technologies, Seattle, WA), according to the manufacturer’s instructions. Each sample was normalized to expression levels of the housekeeping genes, and the geometric mean was used to compute the normalization factor. Any mRNA samples below 20 counts were excluded from analysis. Results were analyzed with nSolver Analysis (v. 4.0) and Advanced Analysis Software (v. 2.0).

## Results

**Figure 1.**
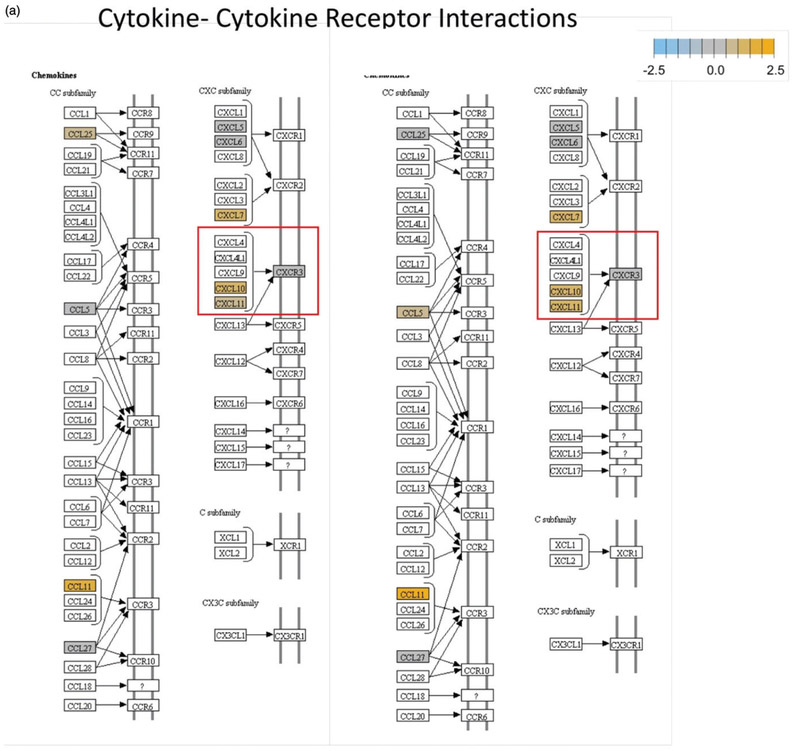

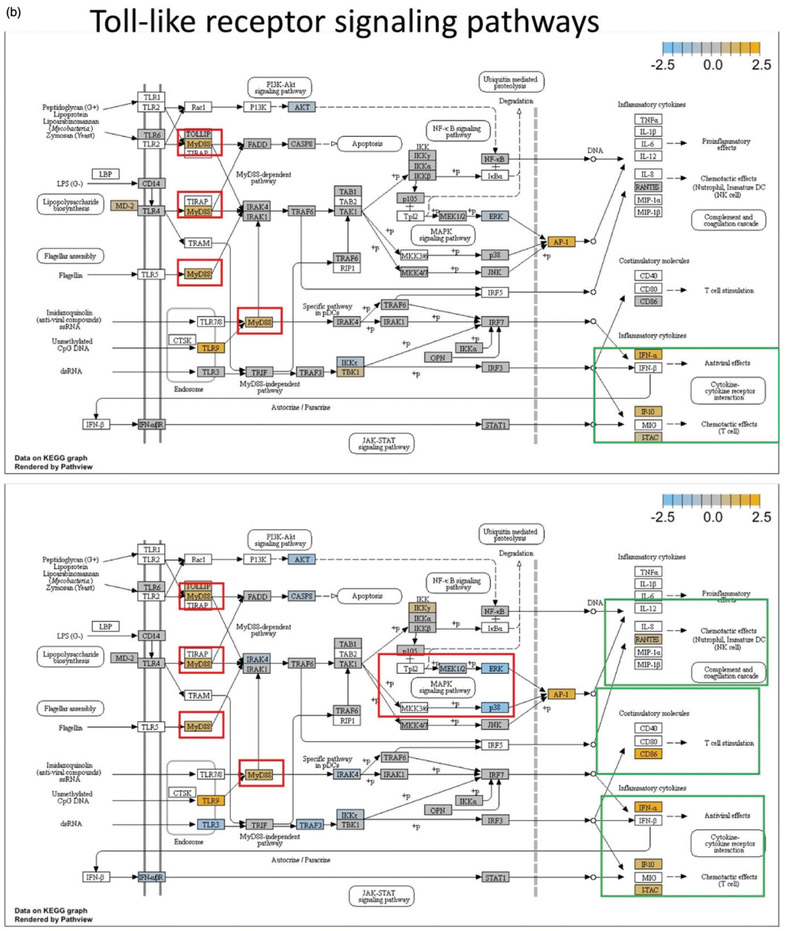
The pathways highlighted (red and green) in the Kegg diagrams demonstrate significant differential expression, compared to control, for a variety of relevant genes following mNPH, radiation or both. The red boxes highlight the genes discussed within the paper, while the green boxes highlight the positive (immune stimulatory, anti-tumor) outcomes of these genetic changes. The diagrams illustrate how mNPH and mNPH + 8 Gy affect each pathway and the possible outcomes or effects of these changes with respect to apoptosis and immune response. The pathways illustrated are (a) the cytokine–cytokine receptor pathway and (b) the toll-like receptor signaling pathway. The comparison also visually demonstrates how the addition of radiation enhances or alters gene expression, compared to low dose hyperthermia alone.

In this study, our primary research findings involve the discovery of immune and cytotoxic based genetic (RNA) and protein changes following the exposure of B16 murine melanoma cells to a single mNPH dose equivalent to 30 min at 43 °C and/or a single dose of 8 Gy. We have demonstrated our genetic finding using classical KEGG pathway diagrams, a heat map and a table format. Semi-quantitative protein results are demonstrated via western immunoblots and a bar diagram. The red and green boxes denoted on the Kegg diagram (Figure 1) demonstrate important specifically altered gene and pathway expression changes.

### Magnetic nanoparticle hyperthermia gene expression

#### mNPH induced mRNA expression

##### Immune pathways.

**Figure 2.**
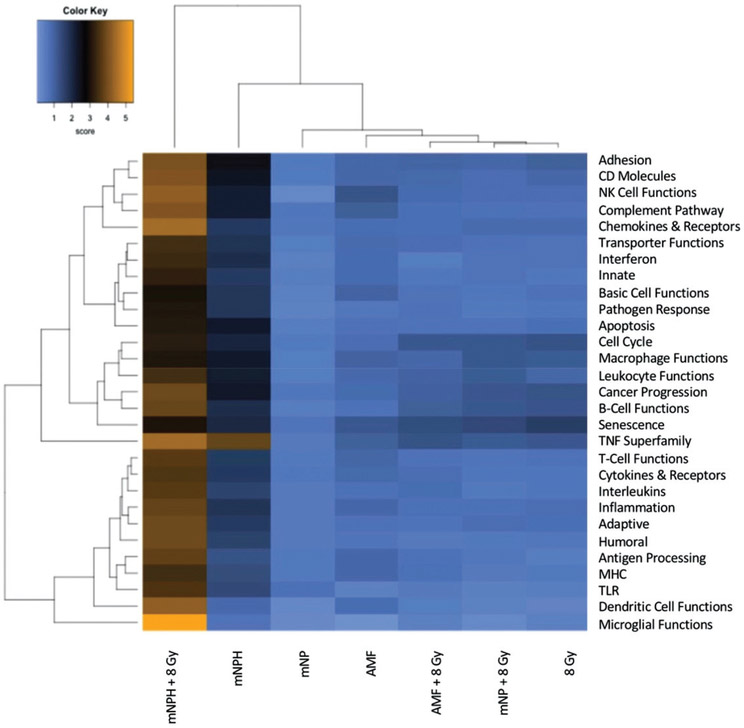
This RNA-based heatmap demonstrates the differences in expression for various gene pathways, following magnetic nanoparticle hyperthermia/mNPH CEM 30 (second column) and a combination of CEM 30 and 8 Gy (first column) as compared to the other treatments including AMF, mNP, AMF + 8 Gy, mNP + 8 Gy and 8 Gy.

Significant genetic changes included selected chemoattractant molecules, and immunogenic, and cytotoxic pathways. These changes are highlighted in Figure 2 in the form of a classical gene expression heatmap. Increased expression changes (yellow) and decreased expression changes (blue) are highlighted for our various treatments across many pathways. Most notable is the marked increased expression of the thermotolerance/immunogenic HSP70 gene [4,5]. Also notable is a minimal change in expression of the chemokine CXCR3 but a significant increase in the expression of its ligands, CXCL10 and CXCL11, which are important in activating other signaling molecules that recruit immune cells for the general enhancement of anti-tumor pathways. The presence of increased CXCL10 and CXCL11 expression without a corresponding increase in CXCR3 suggests mNPH CEM43 30 generates a significant pro-immune genetic expression response (Figure 1). mNPH also generated a decrease in expression of the toll-like receptor pathway signal (TLR3, TLR4) and the associated p38/MAPK pathway. Both pathways are known to be immunosuppressive, pro-tumorigenic, and involved in apoptosis evasion [6-9]. The reduced expression of TLR3, TLR4 and p38/MAPK indicates that low level mNP heating reduces anti-apoptotic and immune suppression signals [10,11]. Changes in the toll-like receptor pathway are demonstrated in Figure 1. Genes differentially expressed in mNPH versus control are colored on the KEGG pathway map demonstrating increases (yellow) and decreases (blue) in genes and their consequences. mNPH CEM43 30 also induced a significant increase in CD86, this gene has been closely associated with immunogenic cell death (ICD).

##### Cell death pathways.

In this study, important members of the Ras/Raf/MAPK pro-survival signaling pathway, ERK1 and ERK2, were downregulated following mNPH, compared to control. Although ERK has both a pro-survival and pro-apoptotic function, depending on the environment, ERK is more likely pro-apoptotic in this setting [9,12,13]. Similarly, the cell death receptor Fas was upregulated following mNPH CEM43 30, indicating a pro-apoptotic response [14]. The DNA repair protein, Gadd45, which plays a role in determining cell survival/apoptosis, was overexpressed following mNPH CEM43 30, also suggesting a pro-apoptotic response [15,16]. Other common pro-apoptotic pathways include the increased expression of several caspase family genes. Our mNPH CEM43 30 RNA results demonstrate lowered expression of CASP8 and CASP3, compared to control, possibly indicating the modest stimulus was too low to initiate the caspase triggered apoptotic activity or that a different apoptotic pathway is activated [14]. A lack of change in PUMA, Bax, Fadd, Bid and GzmA/GzmB, combined with a decrease in Bim, all common pro-apoptotic gene expression patterns, leads to additional questions regarding the robustness of mNPH CEM43 30 to stimulate apoptosis, *in vitro*, at multiple levels.

### Magnetic nanoparticle hyperthermia+radiation gene expression

#### mNPH ± radiation immune related mRNA expression

##### Immune pathways.

As noted, we compared genetic changes for mNPH (CEM43 30), 8 Gy radiation and in combination. Table 1 demonstrates differential expression changes, select group of genes, for all three treatments compared to control. While this study is focused on mNPH and mNPH+radiation, it is important to note that radiation alone did not demonstrate a greater immune or cytotoxic gene enhancement, in any gene or pathway, compared with mNPH+radiation. Compared to mNPH alone, mNPH+radiation significantly increased the expression of notable immune and apoptotic genes such as TLR4, TLR3, CXCL11 and CD86. Expression of the potent immunogenic gene, ICAM1, which codes for a ligand that binds to and activates leukocytes, was not increased following mNPH but was significantly increased following the combined treatment [17,18].

##### Cell death pathways.

Virtually all of the cell death pathways activated by mNPH were activated to a greater extent by the combined mNPH+radiation treatment. Genes such as ERK2, CASP3, MAPK11 (p38b), Fas and PUMA demonstrated significantly enhanced expression. As with mNPH alone, decreases in ERK2 and MAPK11 expression, following mNPH+radiation could demonstrate a reduction in tumor cell survival signaling through the p38/MAPK pathway. Greater increases in PUMA expression, a well-known pro-apoptotic gene, along with an increase in cell death receptor Fas, demonstrate enhanced activation of the apoptotic cascades following mNPH+radiation to activate to a greater degree than mNPH or radiation.

**Figure 3.**
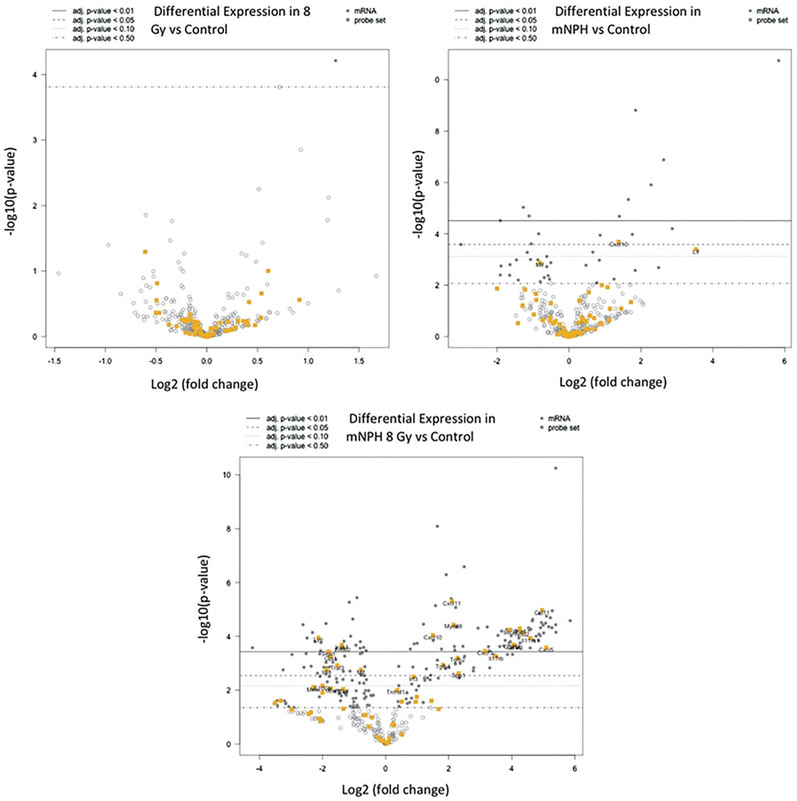
These volcano plots demonstrate differential gene expression fold change following 8 Gy, mNPH or mNPH + 8 Gy, compared to control (on an *x*-axis log2 scale, with the associated *p* values). Volcano plots demonstrate gene expression folds changes in two ways: data points moving to the left or right, from the zero point demonstrate positive or negative expression fold change (circles). The higher the data points rest on the *y*-axis the more statistically significant the change. In this volcano plot, the yellow circles represent those in the cytokine/cytokine receptor pathway. These results suggest that neither 8 Gy nor mNPH alone is a dominant expression promoting factor; rather, the treatments appear to work synergistically together to alter gene expression. The gray circles represent altered genes that not immune or cytotoxicity based. The open circles represent genes who altered expression is not statistically significant at *p*≤.05.

As mentioned previously, the combination mNPH + 8 Gy treatment resulted in a near uniform increase immune and cell death gene expression compared to either single treatment. The first two volcano plots in Figure 3 demonstrate the elevation and significance of gene expression following mNPH and 8 Gy alone. The third plot demonstrates the marked gene expression changes following the combination treatment. Plot 3 demonstrates just how effective a low dose hyperthermia and radiation treatment can be in generating a genetic immune and cell death pathway response.

### Magnetic nanoparticle hyperthermia ± radiation induced protein expression

To verify that the changes in RNA were translated, we examined protein expression in 12 select targets.

**Figure 4.**
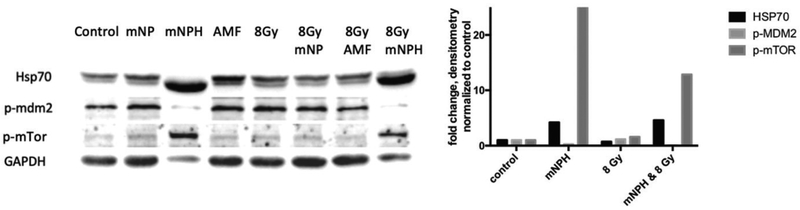
These western/immune blots demonstrate the effect of mNPH CEM 30, 8 Gy and 8 Gy+mNPH had on various protein expression level, with GAPDH as a loading sample. Although mNPH and 8 Gy/mNPH had lower GAPDH than the other conditions, the amount of HSP70 protein was dramatically greater, as was p-mTOR. Additionally, p-MDM2 expression was very significantly decreased following hyperthermia and combinatorial treatments.

Of the 12 targets examined, only three showed differential protein expression; HSP70, p-MDM2 and p-mTOR were markedly over expressed via Western blot and in concern with gene expression. Figure 4 shows a representative Western blot, with densitometry across all blots represented by mean fold expression changes in the corresponding bar graph. HSP70, an important cellular regulator of various types of cell stress, including hyperthermia and immune signaling, demonstrated a 57 × RNA and 3× protein increasing expression, respectively, following CEM43 30, as compared to control. The HSP70 increase following mNPH+radiation was not quite as great as mNPH alone suggesting radiation may slightly mute the response. Both mNPH treatments resulted in an increase in MDM2 gene expression and a decrease in the p-MDM2 protein. p-MDM2 is the activated form of the protein and requires phosphorylation via other proteins, thus an increase in mRNA or total MDM2 protein will not necessarily translate to an increase in the activated (p-MDM2) form. This finding cannot be fully explained from these data; however, the MDM2 gene is associated with p53 negative feedback loop that promotes cell growth and proliferation [19,20], a decrease in p-MDM2 protein expression suggests a tumor suppressing effect mediated through p53. It is important to note that p53 is not mutated in B16F10 murine melanoma cells [21]. mNPH alone and mNPH+radiation had no effect on the genetic expression of mTOR, which has generally been considered an indicator of cell growth and proliferation; however, there was a significant decrease in p-mTOR protein expression following all treatment modalities [12,22].

**Figure 5.**
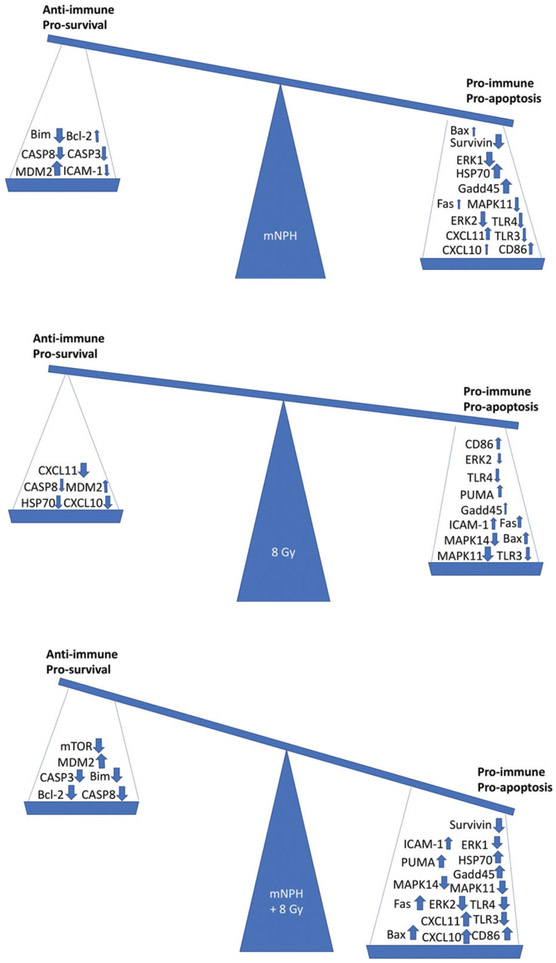
These balances demonstrate the overall effect of the mRNA expression changes for mNPH, 8 Gy and mNPH + 8 Gy as compared to control. The pro-immune/pro-apoptotic versus anti-immune/anti-apoptotic genetic change summary demonstrates the positive effect of mNPH alone, but highlights the much larger effect stimulated by combining mNPH with 8 Gy.

The above discussed results include both positive (pro-immune/pro-apoptotic) and negative (anti-immune/anti-apoptotic) gene expression effects, which are summarized in Figure 5. The balance of these effects is what will determine how effective a therapy is in the long term.

## Discussion

These studies provide quantifiable evidence that a low thermal dose (CEM43 30/thermal dose equivalent to 30 min at 43 °C) generated by mNPH increases the expression of anti-cancer immune and cell death pathway genes and proteins in cultured melanoma cells. A number of the most significant activations, such as cytokine–cytokine receptor pathways including cancer regulating chemokines CXCL10, CXCL11, the T-cell trafficking chemokine CXCR3 [23,24] and the toll-like receptor signaling pathways TLR3/TlR4 are drivers of well accepted anti-tumor or pro-immune response groups. A number of the positively expressed genes and pathways have been demonstrated to directly affect specific anti-cancer immune cell subtypes and, depending on the cell type, could result in different pathophysiological activities. It is also, of course, true that our findings could have different implications in multicell tissues settings. With regard to the apoptosis pathways, our results demonstrate both pro-apoptotic and anti-apoptotic changes are stimulated by mNPH. The balance between these changes, and the function of the genes with altered expression are important in understanding the true effect and balance of the apoptotic pathways.

Our studies also showed that a number of genes involved in injury, repair, survival and immune signaling including Gadd45, Bim, survivin, MDM2, ERK1 and CASP8, are stimulated (altered expression) more by mNPH alone than by 8 Gy or even mNPH + 8 Gy. The changes seen in genes such as ERK1, and survivin, demonstrate how low dose mNPH can effectively activate immune and cell death related pathways, in cancer cells. It is important to note that the expression level of the important immune and cytotoxic ERK1 gene/p-ERK protein was affected differently, by mNPH, depending on the amount of mNPs used in the experiment. If enough mNPs were used to create a measurable thermal dose we found ERK1 expression to be decreased; however, when the identical experiment was conducted, but with fewer mNPs and no measurable thermal dose, an increase in p-ERK protein expression was found.

Although we have not performed genetic validation such as pathway blocks or gene knockout studies, our evidence suggests a number of important potential cancer therapy leads including the TLR3 and TLR4, expressed by tumor cells, may activate cytokine pathways that lead to immune cell recruitment and an inflammatory response via the MyD88 pathway. Interestingly the decreased TLR4 and TLR3 RNA signal seen in our study did not prevent a high expression of MyD88 [6,25]; the down regulation of the p38/MAPK pathway, upregulation of the cell cycle associated gene p21 and the increased Gadd45 expression suggest cell cycle arrest and pro-apoptosis.

Recent studies demonstrating a decrease in p38 and a potential increase in pro-apoptotic PUMA pathway, support our results demonstrating involvement of p38/MAPK pathway following mNPH treatments [10]. Finally, while some of our mNPH and radiation data show a decrease in CASP3 and the possibility of apoptotic pathway suppression, other apoptotic pathways appear, simultaneously, activated.

Finally, it is important to mention again that while these studies contain potentially valuable hyperthermia and radiation immunomodulation and cell death pathway information, without validation in additional cells lines, the data remain in the promising discovery category.

## Figures and Tables

**Table 1.** This table demonstrates differential RNA-based gene expression, in linear fold change, for various immune and/or cytotoxic relevant genes, as compared to control. Several genes including HSP70, MDM2, Gadd45 and CXCL10, were dramatically altered following low dose (CEM 30) mNPH.

Gene	mNPH	8 Gy	mNPH 8 Gy
ICAM-1	.766	1.53	15.7^*^
CD86	2.09	1.32	29.1^*^
CXCL10	2.6*	.657	2.83^*^
CXCL11	1.18*	.713	4.28^*^
CXCR3	1.39	1.31	8.86^*^
TLR4	.81	.864	.457
TLR3	.728	.848	.24^*^
ERK1 (MAPK3)	.478*	1.06	.402^*^
ERK2 (MAPK1)	.837	.882	.564^*^
MAPK11	.619	.838	.2*
MAPK14	1.15	.85	.842
Bcl-2	1.53	1.19	.982
Bax	1.38	1.46*	1.14
PUMA (bbc3)	1.04	1.53	4.75^*^
CASP8	.687^*^	.893	.572^*^
CASP3	.638	.976	.343^*^
Gadd45	3.38^*^	1.21	4.65^*^
Bim (bcl2l11)	.481	1.09	.364^*^
survivin	.373	.923	.1^*^
mTOR	.902	.898	.781
HSP70 (hspab1)	57*	.884	41.8*
MDM2	3.61^*^	1.65*	3.12*
Fas	2.08^*^	1.54	6.85^*^

These expression changes were amplified with the addition of radiation. Other notable genes such as the pro-apoptotic gene bbc3 (PUMA) were altered mostly by the combination treatment.

* Statistically significant at *p* < .05, differential expression.
